# Prescribing-Assessment Tools for Long-Term Care Pharmacy Practice: Reaching Consensus through a Modified RAND/UCLA Appropriateness Method

**DOI:** 10.3390/pharmacy9040194

**Published:** 2021-12-03

**Authors:** João R. Gonçalves, Betsy L. Sleath, Manuel J. Lopes, Afonso M. Cavaco

**Affiliations:** 1 iMed.ULisboa, Social Pharmacy Department, Faculty of Pharmacy, University of Lisbon, 1649-003 Lisboa, Portugal; acavaco@ff.ulisboa.pt; 2 Eshelman School of Pharmacy, University of North Carolina at Chapel Hill, Chapel Hill, NC 27599, USA; betsy_sleath@unc.edu; 3 College of Nursing S. João de Deus, University of Évora, 7000-811 Évora, Portugal; mjl@uevora.pt

**Keywords:** long-term care, potentially inappropriate prescribing, pharmacist, pharmacy practice, consensus, RAND/UCLA, prescribing-assessment tools

## Abstract

Medicines are the most used health technology in Long-Term Care. The prevalence of potentially inappropriate medicines amongst Long-Term Care patients is high. Pharmacists, assisted by prescribing-assessment tools, can play an important role in optimizing medication use at this level of care. Through a modified RAND/UCLA Appropriateness Method, 13 long-term care and hospital pharmacists assessed as ‘appropriate’, ‘uncertain’, or ‘inappropriate’ a collection of commonly used prescribing-assessment tools as to its suitability in assisting pharmacy practice in institutional long-term care settings. A qualitative analysis of written or transcribed comments of participants was pursued to identify relevant characteristics of prescribing-assessment tools and potential hinders in their use. From 24 different tools, pharmacists classified 9 as ‘appropriate’ for pharmacy practice targeted to long-term care patients, while 3 were classified as ‘inappropriate’. The tools feature most appreciated by study participants was the indication of alternatives to potentially inappropriate medication. Lack of time and/or pharmacists and limited access to clinical information seems to be the most relevant hinders for prescribing-assessment tools used in daily practice.

## 1. Introduction

Long-Term Care (LTC) encompasses a range of healthcare, personal care, and other supportive services targeted to patients whose capacity for self-care is limited over an extended period [1,2]. In Portugal, the National Network for Long-Term Integrated Care represents the country’s response to the growing demand for this level of care. Structurally, the National Network for Long-Term Integrated Care (NLTIC) comprehends Home and Community-Based Services (outpatient settings) and Skilled Nursing Homes (inpatient settings). Pharmacists’ intervention in NLTIC outpatient settings is absent. National Network for Long-Term Integrated inpatient settings is divided into ‘Convalescence units’, ‘Medium Term & Rehabilitation units’, and ‘Long-Term & Maintenance units’ [3]. Although not explicitly targeted to care for aged people, LTC populations are often elderly patients. In 2020, 84% of NLTIC’s patients were aged 65 or over, aligned with the worldwide demographic ageing trend [4]. As extensively reported, the ageing process is highly associated with multimorbidity and, consequently, with augmented use of medications [5,6,7]. Older populations are more susceptible to Adverse Drug Events (ADEs) given the age-related pharmacokinetics and pharmacodynamics changes, adding to elders’ usual under-representation in clinical trials. All previous reasons might explain the high prevalence of Potentially Inappropriate Medication (PIM) in older patients [8,9]. A recent systematic review identified a prevalence of inappropriate medication use among the elderly ranging from 11.5% to 62.5% [10]. In LTC facilities, the prevalence of inappropriate medication is also high [11,12,13]. Consequently, the quality of prescribing in LTC populations is of utmost importance, being a complex and challenging process. Potentially Inappropriate Prescribing (PIP) has been associated with negative health outcomes and economic losses [14,15]. Potentially Inappropriate Prescribing concept encompasses the following practices: (i) misprescribing, i.e., the prescription of a medication that could potentially lead to a significant risk of ADEs, due to erroneous posology or route of administration or due to increased risk of drug-drug or drug-disease interaction; (ii) underprescribing or Potential Prescribing Omission (PPO), i.e., the omission of a medication that is clinically indicated for disease treatment or prevention; (iii) overprescribing, i.e., the prescription of medications for which no clear clinical indication exists [16,17]. Different methods have been developed and implemented focused on inappropriate prescribing prevention, such as education, medication reconciliation, and prescribing-assessment tools [18]. Over the last three decades, several criteria and screening tools have been developed to assist clinicians in identifying and preventing PIP. These tools can be explicit (i.e., criteria-based) or implicit (i.e., judgement-based) [19,20]. Amongst the healthcare professionals capable of using such instruments are pharmacists. Pharmacists’ practice encompasses several medicine-related interventions, from medicines management to establishing, assessing, or monitoring treatment plans. Through a set of clinical and patient-oriented activities and collaboration with other healthcare professionals, pharmacists can reduce PIP also in LTC settings [21,22].

Long-Term Care conceptualization and delivery vary from country to country, hampering scientific reporting and standardized utilization of Potentially Inappropriate Prescribing (PIP) assessment tools [23,24]. In Portugal, no common or generally accepted guidelines regarding therapy optimization are known in LTC. The existing heterogeneity of medication prescribing practices, the wide range of supporting instruments available, and the LTC population heterogeneous health status turns challenging to identify the most suitable Prescribing-Assessment Tools (PATs). This study aimed to identify the most suitable Prescribing-Assessment Tools useful for LTC pharmacy practice. An additional goal was to map key characteristics of Prescribing-Assessment Tools from the pharmacist-user perspective and potential hinders for their use.

## 2. Materials and Methods

This study followed a consensus-building methodology to select the most appropriate tools for pharmacy practice in LTC settings, adjusted to patients’ characteristics and pharmacists’ activities in this level of care.

**Figure 1:**
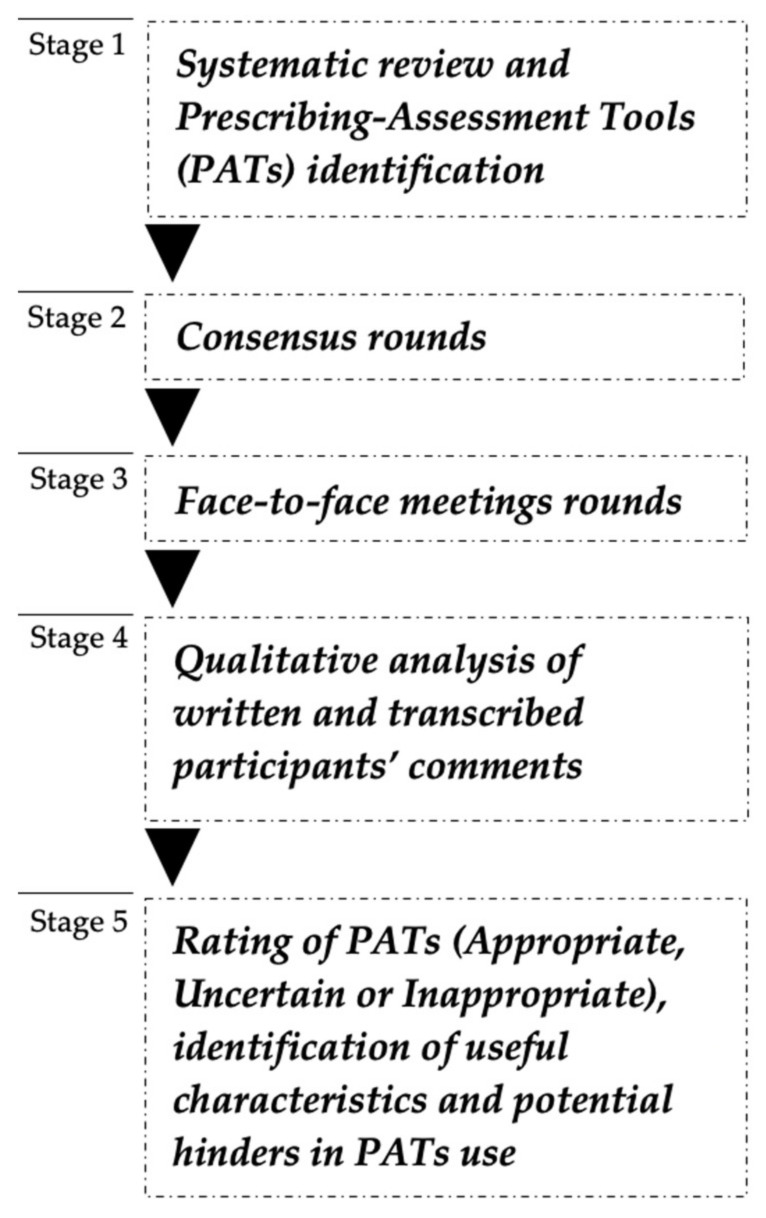
Study methodology flowchart.

The methodological approach is summarized in Figure 1.

Consensus methods can be defined as a set of facilitation techniques designed to explore the level of consensus among a group of experts by synthesizing and clarifying expert opinions. In healthcare research, four consensus methods are frequently used: Delphi, Nominal Group technique, Consensus Development Conference, and RAND/UCLA Appropriateness Method (RAM) [25,26]. RAM is accomplished from a list of predetermined items [27]. For this reason, RAM suits our objectives once Prescribing-Assessment Tools are already developed and published in the scientific literature. Generically, RAM methodology can be described in four main steps: (i) literature review; (ii) panel rating round to review the evidence gathered from literature (without any interactions between panelists, the silent round); (iii) face-to-face meeting aimed at discussing group’s ratings, followed by individual opportunities for re-ratings; (iv) final classification of evidence as “appropriate”, “uncertain” or “inappropriate” (according to panelists median scores) and discussion [27]. A pilot phase was undertaken to anticipate potential hinders in applying this method, particularly for the meeting step (e.g., time and other constraints). At this point, a RAM modification was assumed to strengthen a qualitative perspective on PATs use, inspired by a mixed-methods approach. Instead of a single round, two silent rounds with all panelists were conducted to obtain PATs quantitative appraisal before the face-to-face meeting.

### 2.1. Literature Review and Prescribing-Assessment Tools Identification

Prescribing-Assessment Tools were identified in studies reporting pharmacist-led interventions in institutional LTC settings from published primary sources summarized in a recent systematic review, published elsewhere (Figure 1) [28]. Additionally, a manual search of relevant references from the retrieved papers was undertaken.

### 2.2. Prescribing-Assessment Tools Independent Rating

Panelists’ enrollment was made following a purposive sampling technique (Table 1). Panelists were LTC pharmacists exclusively dedicated to at least one of the three types of RNCCI inpatient settings (‘Convalescence units’, ‘Medium Term & Rehabilitation units’, and ‘Long-Term & Maintenance units’) or hospital pharmacists. Referral of patients to the National Network for Long-Term Integrated Care is made mainly through hospital settings; therefore, the intervention of hospital pharmacists in the transition of patients to LTC occurs. Community pharmacists are not involved in the transition of care; thus, they were not recruited. The final PATs collection was mailed to institutional addresses of each pharmacist for judgment and private rating, as described next.

Panelists were asked to rate each PAT regarding its suitability for LTC pharmacy practice (How appropriate could this PAT be in providing care to LTC patients?), using a 9-points Likert scale (1—totally inappropriate; 9—totally appropriate), and to comment on their ratings. Ratings of 1–3, 4–6 and 7–9 were classified as Inappropriate (I), Uncertain (B), and Appropriate (A), respectively. After each round, the group median rate, Disagreement Index (DI) and comments supporting ratings were anonymously shared with all panelists, who were allowed to change their ratings before the second round and face-to-face meeting.

Disagreement Index is an indicator of consensus and for its calculation, the following equation was used: DI = IPR/IPRAS (IPR, Inter-Percentile Range; IPRAS, Inter-Percentile Range Adjusted for Symmetry), considering the following: (i) IPR = 70th–30th percentile; (ii) IPR Central Point (IPRCP = (70th + 30th percentile)/2; (iii) Asymmetry Index (AI) = (5–IPRCP); (iv) IPRAS = 2,5 + (AI × 1,5) [27]. A DI value less than or equal to 1 signals agreement between panelists [29].

### 2.3. Face-to-Face Meetings

Live remote meetings were held with panelists after the two silent rounds to reach a consensus. An earlier pilot panel, meeting online with 3 LTC pharmacists, took 3 h and 15 min to its end. Thus, to assure that all participants could express their evaluation of all PATs, including qualitative accounts, within an acceptable timeframe, it was necessary to convene three groups of 3–5 panelists. All panels aimed to reach an agreement on assessing PATs as Appropriate, Uncertain and Inappropriate, regarding their usefulness for LTC pharmacy practice. In case panelists disagreed, that panel’s rating was considered as Uncertain (B). For final consensus as Inappropriate (If), Uncertain (Bf), and Appropriate (Af), a thumb rule was followed: for PATs with at least two panels’ rating aligned with the 2nd round overall rating and a DI equal or below 1, final consensus as If, Bf or Af was considered accordingly to the overall group’s rating. If the two panels’ ratings were not aligned with the overall group’s rating, the PAT was classified as Uncertain (B) use for LTC pharmacy practice.

### 2.4. Analysis of Panellists’ Accounts and Final Classification

To inform reasons for ratings and consensus and explore PATs’ characteristics identified as more suitable for pharmacist LTC practice, a record of comments (written during the private rating and transcribed from face-to-face meetings) was performed. The panelists’ accounts were interpreted, organized, and synthesized into two main themes (positive features and usage hinders) without pursuing a detailed qualitative analytical approach.

## 3. Results

### 3.1. Literature Review

**Figure 2:**
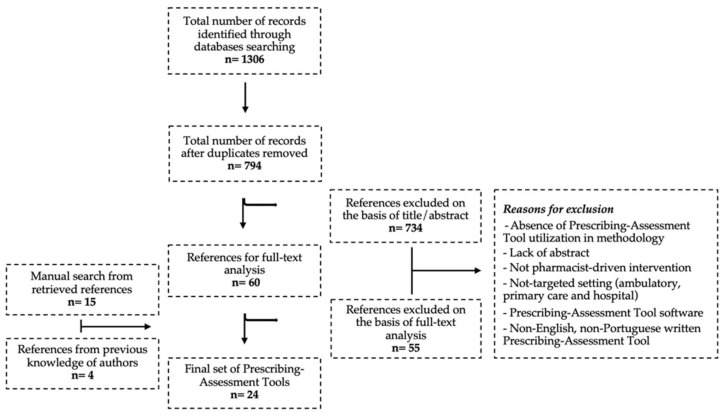
Flowchart of literature review and Prescribing-Assessment Tools identification.

The literature review process (Figure 2) retrieved 24 PATs (Supplementary Table S1). These were organized into different practice areas, namely: (i) anticholinergic and sedative medicines usage: ARS [30] and DBI [31]; (ii) hemorrhagic risk: ATRIA [32], CHA2DS2VASc [33], HAS-BLED [34] and HEMORR2HAGES [35]; (iii) antibiotics usage: Loeb [36]; iv) medication complexity: MRCI [37,38] and Mrs. GRACE [39]; (v) comprehensive implicit assessment of prescribing: MAI [40] and PAI [41]; (vi) comprehensive, explicit assessment of prescribing: Beers criteria [42]; NORGEP [43]; STOPP/START [44]; FORTA [45,46]; Winit-Watjanas criteria [47]; Rancourts criteria [48]; Poudels criteria [49]; McLeods criteria [50]; Laroches criteria [51]; Australian Prescribing Indicators Tool [52] (vii) medicines usage in dementia: APID [53], Holmes criteria [54] and Krogers criteria [55].

### 3.2. Panellists’ Characteristics

Participants’ characteristics are described in Table 1. Participants enrolled included pharmacists working in Long-Term Care Facilities (LTCFs) or hospitals from four Regional Health Authorities (RHAs) catchment areas (out of 5 RAHs in mainland Portugal).

Three meetings with three panelists each were organized. The face-to-face panels were formed by participants 1, 2, 3 (panel 1–LTC pharmacists), 4, 5, 6 (panel 2–LTC pharmacists), 10, 11, 13 (panel 3–Hospital pharmacists), i.e., a dropout rate of 30% was verified (participants 7, 8 and 9).

### 3.3. RAM Rounds, Face-to-Face Meetings and Final Consensus

For 14 PATs, consensus was reached in all components, i.e., overall group rating (with DI ≤ 1) was aligned with the three panels ratings. For instance, in the case of ATRIA, the second-round rating was 7 (A)–with a DI of 0.164–and panels’ 1, 2, and 3 ratings were A; thus, the final consensus was set as Af (Appropriate). For 10 PATs, one panel rating was not aligned with the rest of the ratings. For instance, in the case of DBI, the overall group’s rating was C (Inappropriate) with a DI of 0.438; panels’ 1 and 2 ratings were C, while panel’s 3 rating was B; thus, the final decision was Cf (Inappropriate). For all PATs, DI at the second round was lower than 1. If this rule could not be followed, a PAT was rated for Uncertain (Bf) usefulness in LTC pharmacy practice. Results are summarized in Table 2.

### 3.4. Relevant Characteristics of PATs

Several characteristics of interest emerged from comments and face-to-face meetings qualitative analysis (Table 3).

Four main groups of potential determinants hindering the use of PATs have also been identified through qualitative analysis (Table 4).

## 4. Discussion

This study aimed at identifying useful Prescribing-Assessment Tools for LTC pharmacy practice and, additionally, to map its most relevant characteristics and potential hinders.

LTC pharmacy practice varies regarding the population served and patients’ characteristics (e.g., units exclusively dedicated to cognitive disorders) and pharmacist presence (on-site vs. periodic visits [4,56]. For these reasons, identifying PATs that might be useful for all LTC pharmacists and in the transition of care of LTC patients (hospital-LTC facility) may be difficult. Nevertheless, the RAND methodology allowed to reach a consensus on the most suitable tools for LTC pharmacy practice and mapped its most valuable characteristics. The number and diversity of enrolled participants (e.g., varying years of experience) enriched the discussion and strengthened the potential to translate conclusions into practice. This study is the first to use the RAND approach in LTC pharmacy practice research to the best of our knowledge.

Inappropriate prescribing has been studied and published in the scientific literature for many decades; thus, tools addressing this topic have a considerable number. Kaufmann et al. systematic review identified 46 PATs, of which 6 (13%) were initially developed for LTC use [20]. Similarly, Masnoon et al. identified 42 tools for assessment of the appropriateness of prescribing [19]. To address the apparent scarcity of PATs specifically designed for LTC, we have decided to identify PATs previously used in the LTC context [28]. Additionally, we tried to reduce the set of PATs for panelists’ assessment to avoid loss of richness during meeting discussions.

The 24 PATs were grouped into seven areas: anticholinergic and sedative medicines; hemorrhagic risk; antibiotics; medication complexity; dementia; comprehensive assessment of prescribing (implicit or explicit).

Participants reported anticholinergic and sedative medicines usage and hemorrhagic risk as relevant in the LTC pharmacy context. Scoring systems estimating the risk of bleeding in patients on anticoagulation, such as HAS-BLED and ATRIA, were classified as “Appropriate”, while CHA2DS2VASc and HEMORR2HAGES were classified as “Uncertain”. The difference in classification here seemed to be based on the nature and availability of the data needed to calculate the risk, like CYP 2C9 polymorphisms or previous diagnoses, often not available during the transition of care (e.g., history of alcohol abuse).

Pharmacists working in LTC facilities or hospitals expectably disagree on antibiotic use, knowing antibiotics’ range and use differ in both settings. In RNCCI facilities, antibiotic use is often empirical (i.e., not assisted by antibiogram), while some antibiotics are exclusive of hospital use. In the rating of this particular PAT, hospital and LTC pharmacy practice differences were highlighted, with hospital pharmacists’ panel rating it as “Inappropriate” and both LTC pharmacists’ panels as “Appropriate”. Nevertheless, if considering only LTC practice and excluding transition of care, Loeb’s criteria seem appropriate.

Pharmacists identified some strengths on PATs for medication complexity, yet its usefulness seemed limited. Both MRCI and MRS.GRACE were rated as “Uncertain” for their usefulness for LTC pharmacy practice, mainly given its length (i.e., too time-consuming). However, participants consider these tools as potentially useful at post-discharge moments and to ease medicines administration burden by nurses [57,58].

Prescribing-Assessment Tools targeted to medicines optimization in dementia (APID, Holmes’s criteria, and Kroger’s criteria) were all classified as “Uncertain”. The usefulness of these PATs seems to considerable differ based on pharmacists’ experiences with delivering care to patients with dementia or not. Further research on PATs in dementia is recommended, mainly due to the high prevalence of dementia and cognitive disorders amongst LTC patients [59,60].

Prescribing-Assessment Tools not targeted to a disease/syndrome or a medicines group comprised 50% of all PATs assessed (n = 12 out of 24). PATs used for a comprehensive assessment of prescribing were divided into explicit and implicit. Explicit criteria represent the larger group (n = 10), with Beers’, Laroche’s, and Poudel’s criteria, FORTA and STOPP/START being classified as “Appropriate” for LTC pharmacy practice. Medication Appropriateness Index (implicit PAT) was also assessed as “Appropriate” by participants.

Explicit criteria (including ARS, ATRIA and HAS-BLED) represent 90% of all PATs classified as “Appropriate” (n = 8 out of 9), aligned with healthcare professionals’ preferences reported in the scientific literature [61,62].

Agreement on the rating was reached for all PATs, and the combination of silent ratings and face-to-face discussion allows us to say that the 9 PATs rated as “Appropriate [30,32,34,40,42,44,45,49,51] have the potential to be suitable for LTC pharmacy practice” and during the transition of care of LTC patients. All the 9 PATs address misprescribing, underprescribing and overprescribing practices included in the Potentially Inappropriate Prescribing concept.

In what concerns to helpful characteristics of PATs, the indication of alternatives to PIM seems to be one of the most relevant characteristics along with an organization by medicine, followed by the indication of recommended dose and duration of treatment. However, regarding PAT’s organization (by medicine vs. by disease), further research is needed to explore more clearly participants’ divide: “I prefer a tool that evaluates [medication] by pharmacotherapeutic group and not so much by pathology because when I’m evaluating a prescription, I evaluate medicine by medicine” (P10) vs. “I prefer a disease-oriented PAT” (P11). On the other hand, lack of time and staff (pharmacists) and limited availability or access to clinical information seems to be the most relevant hinders for PATs use in daily practice.

Some limitations of the study can be mentioned: (i) with exception to Beer’s and MRCI, all PATs are not translated to Portuguese language or no previous validation or adaptation to Portuguese LTC context or medicines use, which may have had an impact on panelists’ assessment; (ii) around 40% of PATs under evaluation are targeted to aged people, and LTC is not exclusively targeted to aged people, although, in 2020, 83% of patients assisted in Portuguese LTC network were 65 years old or over [4]; (iii) other published PATs were not included in the final PATs set for analysis once the methodological strategy; (iv) given the heterogeneity of LTC worldwide and that participants work exclusively in Portugal, application of conclusions in international contexts may be hampered.

## 5. Conclusions

Nine Prescribing-Assessment Tools seem to be suitable in assisting clinical pharmacists’ activities targeted to LTC patients. Except for one (MAI), all PATs identified are explicit criteria (ARS, ATRIA, HAS-BLED, Beers’, FORTA, Laroche’s, Poudel’s, and STOPP/START criteria). These Prescribing-Assessment Tools seem to have the potential to be helpful for LTC patients during the transition of care. Three Prescribing-Assessment Tools were classified as Inappropriate for LTC pharmacy practice (DBI, Australian Prescribing Indicators, and NORGEP). Half of Prescribing-Assessment Tools were classified as “Uncertain”, yet further studies should investigate their relevance in particular areas and circumstances of LTC pharmacy practice (e.g., medication regimen complexity or dementia). Explicit criteria gathered the preference of participants, in line with preferences reported in scientific literature. Some important design characteristics of Prescribing-Assessment Tools seem to be related to convenient features of these tools, such as indicating alternatives to Potentially Inappropriate Medication. Future research should investigate the impact of these PATs in relevant endpoints and outcomes for LTC patients (e.g., mortality, hospitalization).

## Figures and Tables

**Table 1:** Panelists’ demographic characteristics.

Participant	Gender	Setting of Professional Activity	Years of Practice
1	Female	Long-Term Care Facility	10
2	Female	Long-Term Care Facility	15
3	Female	Long-Term Care Facility	5
4	Female	Long-Term Care Facility	15
5	Female	Long-Term Care Facility	11
6	Female	Long-Term Care Facility	1
7	Female	Long-Term Care Facility	3
8	Female	Long-Term Care Facility	1
9	Female	Hospital	3
10	Female	Hospital	4
11	Male	Hospital	5
12	Male	Hospital	4
13	Male	Hospital	24

**Table 2:** Prescribing-Assessment Tools ratings, representative quotations and final consensus.

Prescribing-Assessment Tool	Round 1 Median Rating (13 Participants); Disagreement Index	Round 2 Median Rating (13 Participants); Disagreement Index	Face-to-Face Panels Appropriate (A), Uncertain (B) or Inappropriate (C)			Representative Quotations	Appropriate (Af), Uncertain (Bf) or Inappropriate (Cf) for LTC Pharmacy Practice
			Panel 1	Panel 2	Panel 3		
ARS	7; 0.374	7; 0.164	A	A	B	*“It contains many drugs with anticholinergic potential, but in practice, there is not much alternative. We can even tell the physician that these drugs can cause an Adverse Drug Event (ADE), but what is the alternative? In theory, it is important, but in practice, not so much”* P11 *“I find it very useful because most patients take this medication”* P7 *“Can be useful to minimize common ADEs (e.g., falls in the elderly)”* P13	Af
DBI	3; 0.748	2; 0.438	C	C	B	*“is inappropriate given my context“* P4 *“I find it interesting, but in practical terms, it does not materialise into something I can use”* P10 *“I find it interesting because it takes into account the daily dose and defined daily dose and allows for better choices of dosages and promotion of non-pharmacological strategies; focuses on medicines widely used in our aged population”* P13	Cf
ATRIA	6; 0.519	7; 0.164	A	A	A	*“At the level of daily professional practice, the scale is simple to use, all clinical criteria are easily accessed, unlike other similar scales in which personal history or other diagnoses are not always specified in the hospital discharge note”* P5 *“Very practical and straightforward, the existence of a “score”, that is, “a quantifiable value”, makes it much easier to argue with the physicians, when one intends to make medication reconciliation, for instance,”* P12	Af
CHA2DS2VASc	6; 0.519	6; 0.519	B	B	A	*“Easy-to-apply algorithm in my daily practice”* P6 *“This scale may be more difficult to use, once we do not always have access to the personal background of patients”* P5	Bf
HAS-BLED	6; 0.519	7; 0.217	A	A	A	*“Comprehensive, useful, very systematized”* P4	Af
HEMORR2HAGES	5; 0.968	5; 0.519	B	B	B	*“it may be important because it includes the CYP2C9 polymorphisms, although this information is rarely available”* P13 *“Presents pertinent parameters such as genetic polymorphisms and alcohol abuse”* P7 *“I find it a useful algorithm, but it requires data that is not always accessible in my daily professional practice”* P6	Bf
Loeb criteria	6; 1.04	6; 0.652	A	A	C	*“I consider an algorithm very adapted to the LTC reality in order to assess the prescription of antibiotics for the most common infections”* P5 *“It addresses the three main types of infection that we face daily and allows you to screen the appropriateness of antibiotic prescription in a very quick and simple way ”* P1 *“Not useful in a practical context, since clinical conditions are much more complex than the algorithm reflects”* P12	Bf
MRCI	5; 1.70	5; 0.702	B	B	B	*“Although time consuming, very interesting”* P2 *“It is not feasible for a regular use due to its length”* P9 *“Can be useful in the post-discharge moment; however, it is not practical”* P5 *“It would be very useful in my professional practice, since reducing the complexity of the regimens will reduce potential medication errors, increase adherence and reduce costs”* P6	Bf
Mrs. Grace	5; 0.997	5; 0.997	B	B	B	*“Complete and with objective instructions for action”* P10 *“Very interesting; however, I believe its implementation is hindered due to the limited human resources available in LTC”* P4 *“I find it very useful to use it in the planned discharges as a way to adapt the therapeutic regimens individually to the patient and caregivers and as a way to promote adherence to the therapeutic regimen”* P3 *“the algorithm has little practical application since during patient staying it is not always possible to “simplify” therapy due to having a pre-defined drug formulary”* P5	Bf
MAI	7; 0.219	8; 0.219	A	B	A	*“Very adapted to pharmacists. I find it particularly useful for some patients and not for everyone. It allows our participation in multidisciplinary meetings to become more useful. It can be important in deprescribing activities”* P1 *“I find this algorithm very useful and easy to query. However, it is very time-consuming as it is necessary to review each drug at ten different points for each patient”* P6 *“Interesting for therapeutic reconciliation, although these aspects are already taken into account. It can serve more as a guide than for use in practice”* P4	Af
PAI	5; 0.000	5; 0.000	B	C	B	*“There are many questions that we already ask in daily practice”* P5 *“I find it very easy to use, clear, simple; I think it could be a complement to MAI”* P10	Bf
Australian Prescribing Indicators Tool	3; 0.561	3; 0.519	C	C	B	*“Time-consuming, and its use may not always be feasible. Contains very useful information for my technical-scientific development”* P13 *“It is a too lengthy PAT”* P9 *“I consider this PAT impractical to consult due to its organization by statements and not by medications, physiological systems or therapeutic classes”* P6	Cf
Beers criteria	7; 0.292	7; 0.292	A	A	A	*“Quite adequate to the reality of LTC. Although it is the best known among health professionals and this implies that the prescriptions are very much in line with this criterion, I believe its use is fundamental”* P1 *“Very useful, just missing the suggestion of alternative”* P4	Af
FORTA	7; 0.000	7; 0.09	A	A	A	*“Good instrument for medication review in the elderly and based on a diagnosis. Facilitator of doctor-pharmacist and nurse-pharmacist interactions”* P13 *“Very organized and quick and easy to consult”* P2	Af
Laroche criteria	7; 0.000	7; 0.000	A	A	A	*“Very useful and easy to apply in my daily professional practice. Its main advantage is the fact that it presents the reasons for non-suitability and what are the safest alternatives”* P6 *“It has a good compromise between extension, reasons and alternatives”* P3	Af
McLeod criteria	6; 0.217	6; 0.217	A	B	B	*“I appreciate this PAT because it offers alternatives, the risk associated to PIMs and the statistics about the consensus”* P9 *“It is organized by incorrect practice and not by medication; it is not so direct, it is less practical”* P10 *“It can be difficult to consult, but it is useful to suggest therapeutic alternatives to prescribers”* P2	Bf
NORGEP	3; 0.652	3; 0.652	C	C	C	*“It didn’t seem very useful to me due to its sparse coverage”* P4 *“Little information compared to other criteria”* P11	Cf
Poudel criteria	8; 0.292	8; 0.292	A	A	A	*“This PAT is very interesting because it has indications for withdrawal regimens, in addition to therapeutic alternatives”* P2 *“It complements the Beers criteria by presenting suggestions of drug tapering. Moreover, it presents therapeutic alternatives, similar to Laroche’s criteria”* P12	Af
Rancourt criteria	5; 0.851	5; 0.851	B	B	B	*“I find it interesting in terms of posology–duration of treatment and dosage–as a way to sensitize the doctor”* P13 *“It is not very interesting, as it did not suggest alternatives or give guidance, compared to other PATs”* P4 *“It is not totally incomplete because it considers interactions and dosages”* P6	Bf
STOPP/START	7; 0.164	7; 0.164	A	A	A	*“Easy to consult since it is organized by physiological systems. It also has the advantage of allowing the identification of potential prescribing omissions”* P6 *“Complete and with clear indications of actions”* P10	Af
Winit-Watjana criteria	5; 0.968	4; 0.519	B	B	C	*“It summarizes the most significant interactions and adverse reactions, being useful for a consultation”* P5 *“Nowadays listing interactions doesn’t make much sense because we have search engines for interactions”* P10 *“Does not propose alternatives. Just mention MPIs and some interactions”* P13	Bf
APID	5; 0.968	5; 0.968	B	B	B	*“We often do not have data on admission about the patient to answer these questions”* P6 *“It should include situations of exacerbation (e.g., delirium)”* P12	Bf
Holmes criteria	5; 0.851	5; 0.851	B	B	B	*“Informative and user-friendly”* P10 *“Very little information […] depends on each case”* P4	Bf
Kroger criteria	6; 0.519	6; 0.376	B	B	A	*“It is an adequate PAT because it offers some justification and rationale for the use of medication”* P9 *“More interesting for technical development than for practical application”* P4	Bf

**Table 3:** Prescribing-Assessment Tools’ useful characteristics and representative quotations.

Prescribing-Assessment Tools’ Useful Characteristic	Representative Quotations
Levels of evidence	*[FORTA] because it is organized by level of evidence* (P7) *[…] having the advantage of presenting the level of evidence* (P8, on Beers’)
Dose and duration of treatment	*I consider it a very complete and valuable criterion as it considers factors such as dose and duration of treatment* (P8 on Rancourt’s) *I find it very interesting because it considers daily dose and allows to choose better posology* (P13, on DBI’s) *Useful because it gives a maximum of days, and many times physicians want to use antibiotics more days than those preconized* (P3, on Loeb’s)
Scoring system	*[…] a “score”,* i.e., *a quantifiable value, greatly facilitates discussion with physicians when delivering therapeutic reconciliation, for example* (P12, on ATRIA’s)
Reasons for PIM classification	*Contains very useful information such as reasons for PIM classification* (P13, on Laroche’s) *Presents the reasons why the medicine is potentially inappropriate* (P8, on Beers’)
Inclusion of alternatives to PIMs	*Very useful, only the suggestion of alternatives is missing* (P4, on Beers’) *suggests therapeutic alternatives* (P1, on Laroche’s) *It is a helpful PAT because it suggests therapeutical alternatives* (P9, on Laroche’s) *[…] suggests therapeutical alternative […]* (P5, on McLeod’s) *[…] it offers therapeutical alternatives […]* (P9, on McLeod’s) *[…] presents therapeutical alternatives, similarly to Laroche’s* (P12, on Poudel’s) *It does not suggest alternatives; just mention PIMs and interactions* (P13, on Winit-Watjana’s) *[…] a helpful PAT should include therapeutical alternatives* (P3, McLeod) *Contains valuable information such as alternative medicines* (P13, on Laroche’s) *It suggests therapeutical alternatives* (P13, on Poudel’s) *It does not have alternatives; I consider it a “lower version*” (P12, on Winit-Watjana’s)
Organised by medicines groups	*I consider this criterion more complex to consult because it is organized by inappropriate practice and not by medicine, therapeutic indication, or physiological system* (P8, on McLeod’s) *I prefer a tool that evaluates by pharmacotherapeutic group and not so much by pathology because when I’m evaluating a prescription, I evaluate medicine by medicine and, if a question arises, it will help me to have a PAT by the pharmacotherapeutic group to consult and preferably, with alternatives in case you find something wrong* (P10) *A PAT organized by pharmacotherapeutic groups helps more; it makes it simpler* (P13) *It seemed to be the most organized as it is divided into therapeutic groups* (P3, on McLeod’s) *Easy to consult as it is organized by therapeutic groups* (P2, on Poudel’s)
Inclusion of risk associated with PIM	*I find this PAT interesting because it mentions the risk to the patient* (P5, on McLeod’s) *It includes the risk to the patient […]* (P9, on McLeod’s) *It has the risk associated with the patient* (P3, McLeod’s)
Organised by disease/syndrome	*I prefer a disease-oriented PAT* (P11) *It includes the most prevalent diseases on the LTC national network* (P1, on FORTA’s)
Consensus on PIM/alternative	*It includes the panel agreement on the PIM alternative* (P5, on McLeod’s) *It includes statistics about the consensus on the use of a particular medicine* (P9, on McLeod’s)
Withdrawal regimens	*It has indications on how to withdraw the PIM, being a differentiating factor from the other PATs* (P5, on Poudel’s) *In a way, it complements the Beers’ criteria, as it suggests withdrawal* regimens (P12, on Poudel’s) *It suggests withdrawal regimens* (P13, on Poudel’s)

**Table 4:** Potential determinants hindering Prescribing-Assessment Tool use.

Potential PATs Use Hinders	Representative Quotations
Lack of time / Lack of pharmacists	*Challenging to use considering the low human resources ratio in LTC* (P4, on DBI’s) *I think this PAT would be very useful […] The main limitation is related to the time spent in its application* (P8, on Mrs. Grace’s) *I consider it very useful in my daily professional practice as it is complete […] however, it is a very time-consuming PAT to apply as it is necessary to review each medicine of each patient at ten different points* (P8, on MAI’s) *The amount and turnover of patients does not allow us to look detailed at every prescription using some of these PATs* (P1)
Assistance to several units	*It is not easy to apply in my daily professional practice because it involves discussing these situations with the clinical team when prescribing, and I am not present full time at the facilities* (P8, on Loeb’s) *It is not possible to use it for all prescriptions since there are daily therapeutic changes in the various facilities that I assist, and most of the time, I’m not at the facilities when new prescriptions occur* (P3, on PAI’s)
Communication barriers with the healthcare team	*Despite the quality and usefulness of PATs, the difficulty can come from the physicians because they hardly listen to the pharmacist’s opinion* (P2)
Limited access or availability of information	*We often do not have information to use this PAT because the physician usually does not register this data, and we do not have access to the entire clinical file* (P13, on Loeb’s) *We often don’t have access to patients’ background or is not even available from the transition of care* (P5, on HAS-BLED’s/ HEMORR2HAGES’) *Sometimes I don’t have access to some clinical data (e.g., liver function tests)* (P8, on HAS-BLED’s/HEMORR2HAGES’)

## Supplementary Materials

The following are available online at https://www.mdpi.com/article/10.3390/pharmacy9040194/s1, Table S1: Summary of the identified Prescribing-Assessment Tools.
