# Supplementary material for: Prescribing-Assessment Tools for Long-Term Care Pharmacy Practice: Reaching Consensus through a Modified RAND/UCLA Appropriateness Method
Source: Pharmacy (Basel). 2021 Dec 3;9(4):194. doi: 10.3390/pharmacy9040194 (PMC8708836; doi:10.3390/pharmacy9040194)
Supplement: Supplementary file 1 [file pharmacy-09-00194-s001.zip › pharmacy-1487418-supplementary (1).pdf]

Table S1. Summary of the identified Prescribing-Assessment Tools

Prescribing-Assessment Tools	Authors (year of first publication)	Brief description (of most updated version)
Beers criteria	Beers, M. (1991)	List of Potentially Inappropriate Medications (PIMs) for adults 65 years and older. Medicines recommendations are organized according to i) PIM due to medicines adverse effects profile; ii) PIM due to drug-disease or drug-syndrome interactions that may exacerbate the disease or syndrome; iii) medicines to be used with caution in older adults; iv) PIMs due to drug-drug interactions; v) medicines that should be avoided or used with caution with varying levels of kidney functions; vi) PIMs due to anticholinergic potential.
Anticholinergic Risk Scale (ARS)	Rudolph et al. (2008)	Medicines with anticholinergic properties are scored on a 3-point scale (with 3 comprising medicines with the highest anticholinergic potential). Medicines with no or low anticholinergic risk are scored 0. The ARS score for a patient is the sum of points for each medication. The higher is the score, the higher is the risk of anticholinergic adverse events (e.g. cognitive dysfunction and delirium)
Anticoagulation and Risk Factors in Atrial Fibrillation (ATRIA) Risk Score	Fang, M. et al. (2011)	A 5-items risk score to predict warfarin-associated hemorrhage in patients with atrial fibrillation - anemia (3 points); severe renal disease (e.g., glomerular filtration rate < 30 ml/min or dialysis-dependent (3 points); age ≥ 75 years (2 points); prior bleeding (1 point); and hypertension (1 point).
Appropriate Psychotropic drugs use In Dementia (APID)	van der Spek, K. et al. (2015)	An instrument for assessing the appropriateness of Psychotropic Drug Use (PDU) for neuropsychiatric symptoms in patients with dementia. PDU is evaluated by a seven-item index - indication, evaluation, dosage, drug-drug interaction, drug-disease interaction, duplication, and duration of therapy. Each item is scored from 0 (recommended) to 2 (inappropriate).
Australian Prescribing Indicators Tool	Basger, B. et al. (2008)	A list of 48 prescribing indicators for commonly occurring conditions in patients aged > 65 years, comprising appropriate and inappropriate pharmacotherapy choices.
CHAD2DS2-VASc	Lip, G. et al (2010)	A tool for predicting stroke risk in a patient with atrial fibrillation. Risk estimation is reached by a point-based scoring system - Congestive heart failure/Left ventricular dysfunction, 1; Hypertension, 1; Age ≥ 75, 2; Diabetes mellitus, 1; Stroke/Thromboembolism, 2; Vascular disease, 1; Age 65-74, 1; Sex category (female gender), 1.
Drug Burden Risk (DBI)	Hilmer, S. et al (2007)	A pharmacological-based measure of exposure to medication with anticholinergic and sedative properties, associated with decreased function in the elderly.
Fit for The Aged (FORTA)	Wehling, M. et al (2008)	A list of 190 medications/medications groups commonly used in older patients, whose utilization is classified in 4 classes: Class A (Absolutely); Class B (Beneficial), Class C (careful), Class D (Do not "use"). Medicines are grouped according to disease/diagnosis.
HAS-BLED	Pisters, R. (2010)	A tool for bleeding risk assessment of patients with atrial fibrillation. Risk estimation is reached by a point-based scoring system: hypertension, 1; abnormal renal/liver function 1 or 2; stroke, 1; bleeding history or predisposition, 1; labile international normalized ratio [INR], 1; age ≥ 65years, 1; drugs/alcohol concomitantly, 1 or 2.

Prescribing-Assessment Tools	Authors (year of first publication)	Brief description (of most updated version)
HEMORR2HAGES	Gage, B. et al. (2006)	A tool for bleeding risk assessment of patients with atrial fibrillation. A point-based scoring system reaches risk estimation: history of bleeding, 2; hepatic or renal disease, 1; ethanol abuse, 1; malignancy, 1; age ≥ 75 years, 1; reduced platelet count or function, 1; hypertension (uncontrolled), 1; anemia, 1; genetic factors, 1; excessive fall risk, 1; stroke, 1
"Holmes' criteria"	Holmes, H. et al. (2008)	A list of 69 medication classes stratified as "Always appropriate"; "Sometimes appropriate"; "Rarely appropriate" or "Never appropriate" for use in patients with advanced dementia
"Kroger's criteria"	Kroger, E. et al. (2015)	A list of 63 medications or medication classes stratified as "generally", "sometimes," or "rarely appropriate" for use in nursing home patients with severe dementia.
"Laroche's criteria"	Laroche, ML. et al. (2007)	A list of 34 medication or medication classes stratified as with "unfavorable benefit/risk ratio", with "questionable efficacy" and with both "unfavorable benefit/risk ratio and questionable efficacy" for use in people aged ≥75 years
"Loeb's criteria"	Loeb, M. et al. (2001)	A set of criteria for antibiotic use in Long-Term Care facilities for skin and soft-tissue infections, respiratory infections, urinary infections, and fever when the original focus of infection is unknown
Medication Appropriateness Index (MAI)	Hanlon, J. et al. (1992)	MAI is a 10-item index for medication regimens appropriateness evaluation. Each question, respective to a single medication, is weighted from 0 to 3, namely: Indication, 3; Effectiveness, 3; Dosage, 2; Directions, 2; Practicality, 1; Drug-drug interaction, 2; Drug-disease interaction, 2; Unnecessary duplication, 1; Duration, 1; Expensiveness, 1, with 0 meaning the best score per item. For the final patient's score, the individual scores per medication are summated
"McLeod's criteria"	McLeod, P. et al. (1997)	A list of 71 inappropriate prescribing practices for elderly patients, with its clinical significance, rated from 1 (not significant) to 4 (highly significant) and recommended alternatives. Prescribing practices set is divided into three classes: "drugs generally contraindicated for elderly people", "drug-disease interactions", and "drug-drug interactions."
Medication Regimen Complexity Index (MRCI)	George, J. et al. (2004)	Instrument comprised of 65 items, divided into three components - dosage formulations, dosing frequency, and additional administration directions – to measure the complexity of pharmacotherapy. Each item is scored (weighing from 0.5 to 12.5), and the final complexity index is achieved by adding the scores of the three components. For scores ≥ 13.5, complexity is considered high. The MRCI score calculated for each patient was based on three separate components of their medication regimen: (i) dosage formulations, (ii) dosing frequency, and (iii) additional administration directions
Medication Regimen Simplification Guide for Residential Aged CarE (MRS GRACE)	Chen, E. et al. (2018)	Implicit tool comprised of 5 questions about i) the patient; ii) regulatory and safety requirements; iii) drug interactions; iv) formulation; and v) facility and follow-up consideration, targeted to simplify therapeutical regimen in aged care.

Prescribing-Assessment Tools	Authors (year of first publication)	Brief description (of most updated version)
The Norwegian General Practice Criteria – NORGEP	Rognstad, S. et al (2009)	Explicit criteria for inappropriate prescribing assessment in general practice on people ≥ 70 years. Criteria refer to single drugs/dosages and drug combinations to be avoided for their clinical relevance.
Prescribing appropriateness indicators – PAI	Cantrill, J. et al. (1998)	Implicit criteria encompassing a set of 14 indicators for prescribing assessment of long-term medication
"Poudel's criteria"	Poudel, A. et al. (2016)	Explicit criteria of potentially inappropriate medications and a methodology for appropriate deprescribing of medication commonly used in the elderly.
"Rancourt's criteria"	Rancourt, C. et al. (2004)	Explicit criteria encompassing a total of 111 potentially inappropriate prescribing scenarios, namely: i) medication (n= 39); ii) duration (n= 15), iii) dosage (n= 20), and iv) drug-drug interaction (n= 37).
STOPP/START criteria	Gallagher, P. et al. (2008)	Explicit criteria combining scenarios of potentially inappropriate medicines (Screening Tool of Older People's Prescriptions - STOPP) and potentially prescribing omissions (Screening Tool to Alert to Right Treatment (START). In total, the criteria are comprised of 114 items (80 STOPP criteria and 34 START criteria)
"Winit-Watjana's criteria"	Winit-Watjana, W. et al. (2007)	Explicit criteria comprised of 77 statements, divided into 3 sections: i) adverse reactions; ii) drug-disease interactions, and iii) drug-drug interactions
